# A Case Series of Valve‐in‐Valve Transcatheter Aortic Valve Replacement in Stentless Bioprosthetic Valves Using Self‐Expanding Platform With Minimalist Approach

**DOI:** 10.1002/ccd.31625

**Published:** 2025-06-02

**Authors:** Adeogo Akinwale Olusan, Raj Rajendra, Elved Roberts, Jan Kovac

**Affiliations:** ^1^ Department of Cardiology Glenfield Hospital Leicester UK; ^2^ University of Leicester Leicester UK

**Keywords:** aortic valve degeneration, minimalist approach, self‐expanding valve, stentless bioprosthetic valve, TAVR outcomes, transcatheter aortic valve replacement, valve‐in‐valve

## Abstract

Valve‐in‐valve (ViV) Transcatheter aortic valve replacement (TAVR) has emerged as a valid treatment option in symptomatic patients with failed aortic bioprosthetic valves, most especially in those with intermediate, high or prohibitive risk of surgery. Due to the unique design of stentless bioprosthetic valves with absence of visible posts and stent frame, they present with challenges during ViV TAVR procedure. Our case series assessed the safety, feasibility, and early outcomes of ViV TAVR in patients with failed bioprosthetic aortic valve replacement (AVR) using self‐expanding valve platform (Acurate Neo 2/AN2 valve) between March and September 2024 with minimalist approach. Procedural success, complications and early outcomes (mortality and functional status) were evaluated. A total number of three patients, mean age 75 ± 7 years, 67% female were included in the case series with high Society of Thoracic Surgeons (STS) and European System of Cardiac Operative Risk Evaluation II (EuroSCORE II) score. The mean time from initial surgery to ViV procedure was 18 ± 1 years. ViV procedure was successfully completed in all patients with no major complication, and no more than trivial trans/paravalvular regurgitation on discharge. There was no requirement for permanent pacemaker implantation, no stroke or mortality at 30‐day and there was significant improvement in functional class alongside symptomatic improvement. We report our experience in ViV TAVR using AN2 valve in Shelhigh supra stentless aortic bioprosthesis and that this procedure despite anatomical and procedural challenges is feasible and safe.

## 1 Introduction

Stentless bioprosthetic aortic valves are commonly used in patients requiring AVR, particularly in younger, low‐risk patients because of more favorable haemodynamic profiles [1, 2, 3]. However, structural degeneration leading to their failure poses a significant clinical challenge. Nevertheless, Trans‐catheter aortic valve replacement (TAVR) is a viable treatment of choice in patients with symptomatic severe aortic stenosis (AS) across wide spectrum of surgical risks (inoperable‐high‐moderate‐low risk) [4, 5, 6]. Valve‐in‐valve (ViV) TAVR has emerged as a valid treatment option in patients with failed aortic bioprosthetic valves, most especially in those with intermediate, high or prohibitive risk of surgery [7, 8].

ViV TAVR has been well established for stented bioprosthetic aortic valves, however, there is limited evidence regarding its use in Shelhigh supra stentless aortic bioprosthesis. The absence of visible posts and stent frame makes deployment of the new valve technically challenging moreover, advance adequate planning is very crucial for procedural success. Technological advancement in ViV TAVR in conjunction with better operators' experience have enhanced procedural safety, improved outcomes and decreased complications.

## 2 Case Series Report

We report on three patients aged 68 year old female, 72 year old male and 83 year old female who presented to our tertiary center with decompensated heart failure symptoms according to New York Heart Association class IV (NYHA class IV), NYHA class IV and NYHA class II–III respectively between March and September 2024. Patient 1 had a prior aortic valve replacement (AVR) using a 25 mm Shelhigh supra stentless valve along with wrapping of ascending aorta using Dacron tube in March 2007 for Bicuspid aortic stenosis. Patient 2 had a prior AVR in 2006 using 25 mm Shelhigh supra stentless valve for aortic stenosis and Patient 3 had prior AVR in 2005 using 23 mm Shelhigh supra stentless valve for aortic stenosis. Full detail about their past medical history and medication history can be found in Table 1.

**Table 1:** Baseline patient characteristics.

	Patient 1	Patient 2	Patient 3
Gender	Female	Male	Female
Age	68	72 years	83
Relevant past medical history	AVR and wrapping of ascending aorta with 28 mm Dacron tube in March 2007 for Bicuspid aortic stenosis, Chronic residual Type A dissection due to aortic wrap surgery, Moderate transvalvular AR on TOE in 2023, Arterial hypertension, Hypercholesterolaemia, Chronic kidney disease stage 3, Carpal tunnel syndrome, Moderate MR and TR, Pulmonary hypertension (PASP 60 mmHg), Ex‐smoker	AVR in 2006, Transient ischaemic attack (TIA), Arterial hypertension, Benign prostatic hyperplasia (BPH), Colonic diverticulosis, Small polyps	AVR in 2005, Arterial Hypertension, Chronic rhinitis with postnasal drip, Gastro‐oesophageal reflux disease (GORD)
Medications	Aspirin 75 mg OD Bisoprolol 5 mg OD Ramipril 10 mg OD Furosemide 40 mg BD Atorvastatin 20 mg ON Cinnarizine 15 mg TDS Omeprazole 20 mg OD	Rosuvastatin 10 mg OD Aspirin 75 mg OD Omeprazole 20 mg OD AdCal D3 Diclofenac Sildenafil Dapagliflozin 10 mg OD Bisoprolol 1.25 mg OD Furosemide 40 mg BD Ramipril 1.25 mg OD	Amiloride 5 mg OD Atorvastatin 20 mg OD Candesartan 8 mg OD Clopidogrel 75 mg OD Dymista nasal spray Esomeprazole 20 mg OD Fexofenadine 180 mg Furosemide 80 and 40 mg Spironolactone 12.5 mg OD Doxazosin 2 mg OD
Presentation	Decompensated heart failure (refractory)	Decompensated heart failure	Exertional breathlessness
Shelhigh valve size (mm)	25 mm	25 mm	23 mm
Body composition	Height 155 cm Weight 93 kg BMI 38.7 kg/m²	Height 174 cm Weight 64 BMI 21.1 kg/m²	Height 154 cm Weight 78 kg BMI 32.9 kg/m²

**Figure 1:**
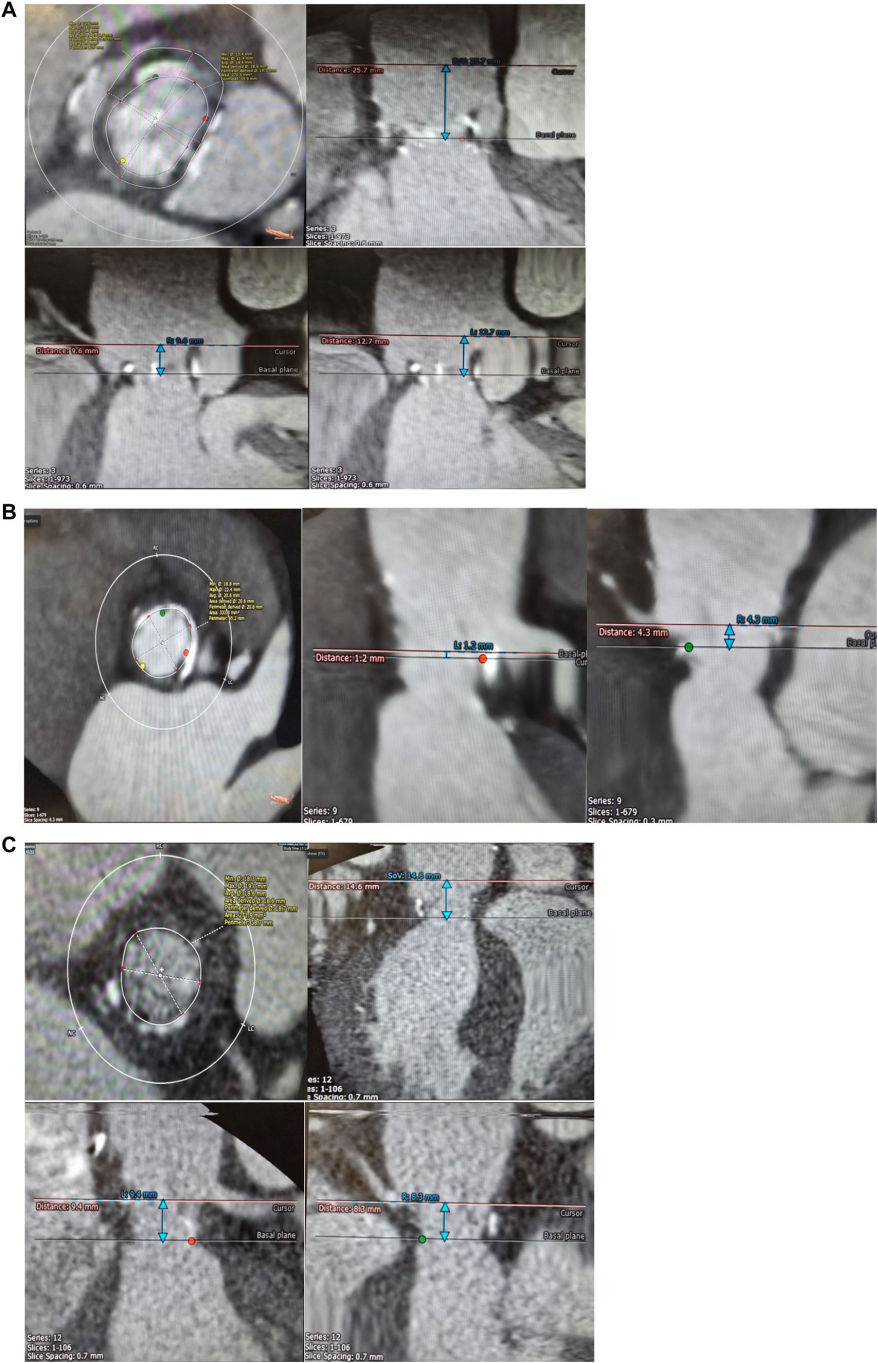
(A) Showing CT scan 3mensio reconstruction of annulus for patient 1. (B) Showing CT scan 3mensio reconstruction of annulus for patient 2. (C) Showing CT scan 3mensio reconstruction of annulus for patient 3. [Color figure can be viewed at wileyonlinelibrary.com]

Patient 1 had initially presented to a local district general hospital in February 2024 with refractory heart failure requiring very high dose of intravenous diuretics (360 mg/24 h of i.v Furosemide) due to severe aortic regurgitation before her transfer to our center. Patent 2 presented to our tertiary center with heart failure symptoms due to mixed aortic valve disease also required intravenous diuretics whereas Patient 3 presented with progressive exertional breathlessness over few months due aortic regurgitation. As a result of their previous surgical histories, they were deemed at high risk for redo‐surgery by the multidisciplinary team (Heart Team) including cardiothoracic surgeons due to high Society of Thoracic Surgeons (STS) score and European System of Cardiac Operative Risk Evaluation II (EuroSCORE II) (Table 2). Hence, they were worked up for ViV TAVR using computerized tomography scan (CT scan) along with 3mensio reconstruction of both annulus (Figure 1a–c) and peripheral arterial system which demonstrated that they were all feasible for transfemoral approach. We intended for minimalist approach using local anaesthesia ± mild sedation, simplified secondary arterial access via radial artery where possible, pacing on left ventricle (LV) wire and post procedural monitoring was streamlined, focusing on clinical assessment and transthoracic echocardiography.

**Table 2:** Preprocedural and procedural characteristics.

	Patient 1	Patient 2	Patient 3
Mechanism of failure on echocardiography	Severe AR, Vmax 3.4 m/s, peak gradient 47 mmHg, PHT 124 ms	Severe AS and AR, Vmax 4.1 m/s, peak gradient 66 mmHg, mean gradient 36 mmHg, AVA 1.2 cm²	Moderate‐Severe eccentric AR, Vmax 2.6 m/s, peak gradient 27 mmHg, mean gradient 15 mmHg, PHT 218 ms with flow reversal in aortic arch
TAVR CT analysis	Annulus 15.4 × 21.4 mm, Area 270 mm², Perimeter 59.9 mm SoV 30.3 mm STJ height 25.7 mm Coronary heights LCA 12.7 mm, RCA 9.6 mm CFA minimum size Right 7.0 mm, Left 6.7 mm	Annulus 18.8 × 22.4 mm, Area 333.3 mm², Perimeter 65.2 mm Coronary heights LCA 1.2 mm, RCA 4.3 mm CFA minimum size Right 8.9 mm, Left 7.2 mm	Annulus 18.3 × 19.0 mm, Area 271.9 mm, Perimeter 58.7 mm STJ height 14.6 mm Coronary heights LCA 9.4 mm, RCA 8.3 mm CFA minimum size Right 6.6 mm, Left 6.7 mm
STS score (%)	15.8	2.16	5.53
EuroSCORE II (%)	20.14	21.86	8.95
NYHA class	IV	IV	II–III
LVEF (%)	30–35	30–35	> 55
NT‐ProBNP (ng/L)	29,413	—	2100
Renal function (pre TAVR)	Creatinine 111 (ⴎmoles/L) eGFR 44 (mL/min/1.73 m²)	Creatinine 134 (ⴎmoles/L) eGFR 45 (mL/min/1.73 m²)	Creatinine 112 (ⴎmoles/L) eGFR 39 (mL/min/1.73 m²)
Vascular access	Right CFA 14 F sheath Left CFA 7 F sheath	Right CFA 14 F sheath RRA 6 F sheath	Right CFA 14 F RRA 6 F
Coronary protection	No	Yes, EBU 3.5 guide, Sion Blue wire, undeployed 4.0 × 28 XIENCE stent	No
Predilation	No	No	No
TAVR size (mm)	AN2 size 23	AN2 size 23	AN2 size 23
Post‐dilation	18 mm true balloon	No	18 mm nucleus balloon
Vascular access closure	Right CFA—2 Proglides Left CFA—1 Proglide	Right CFA—1 Proglide and 8 F Angioseal RRA—TR band	Right CFA—2 Proglides RRA—TR band
Predischarge echo findings	No AR/pVR Vmax 2.42 m/s, Peak gradient 23.3 mmHg, Mean gradient 10.5 mmHg, AVA 1.1 cm², DVI 0.34 LVEF 40% No pericardial effusion	Trivial pVR Vmax 3.52 m/s, Peak gradient 49.4 mmHg, Mean gradient 29.4 mmHg, AVA 1.02 cm², DVI 0.36 LVEF 40%–45% No pericardial effusion	No AR/pVR Vmax 2.57 m/s, Peak gradient 26.4 mmHg, Mean gradient 10.5 mmHg, AVA 1.0 cm², DVI 0.39 LVEF > 55% No pericardial effusion
Renal function post TAVR	Creatinine 78 (ⴎmoles/L) eGFR 67 (mL/min/1.73 m²)	Creatinine 118 (ⴎmoles/L) eGFR 52 (mL/min/1.73 m²)	Creatinine 101 (ⴎmoles/L) eGFR 44 (mL/min/1.73 m²)
NYHA class at 6 weeks post TAVR	I	I	II

**Figure 2:**
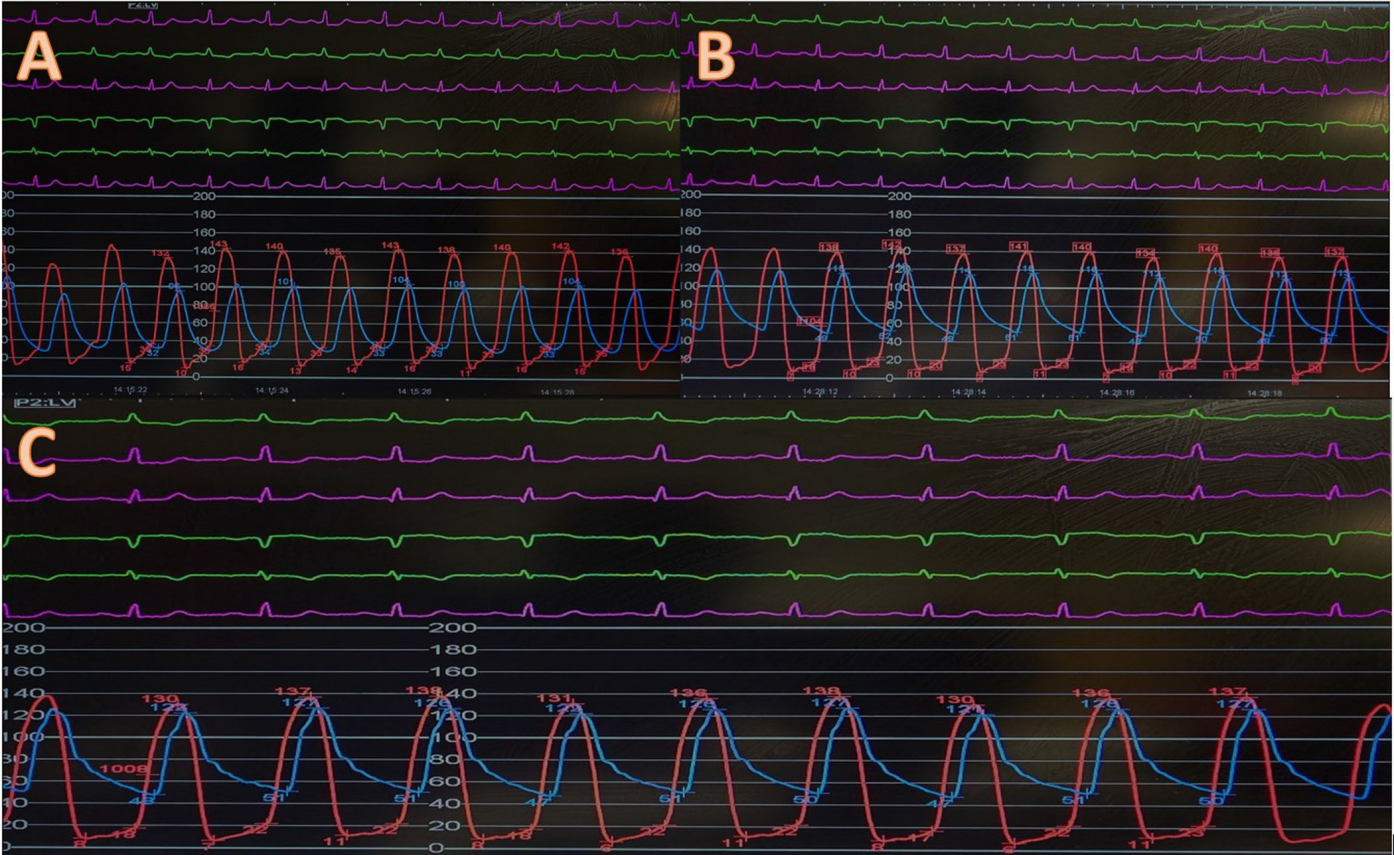
Showing pre and post TAVR invasive Ao/LV pressure measurement for patient 1. (A) Pre TAVR (B) Post TAVR (C) Following postdilation using 18 mm true balloon. [Color figure can be viewed at wileyonlinelibrary.com]

**Figure 3:**
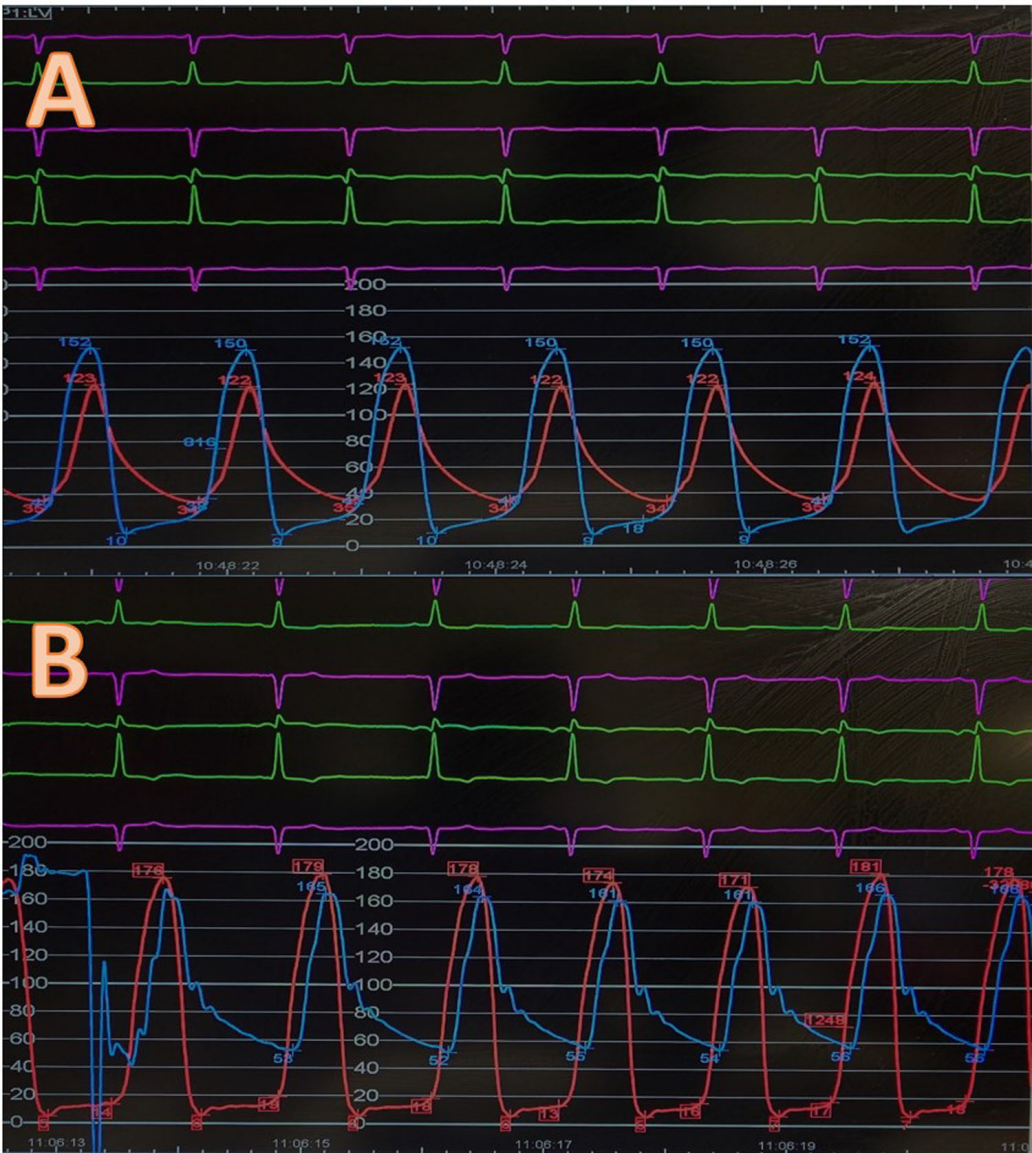
Showing (A) pre and (B) post TAVR invasive Ao/LV pressure measurement for patient 2. [Color figure can be viewed at wileyonlinelibrary.com]

**Figure 4:**
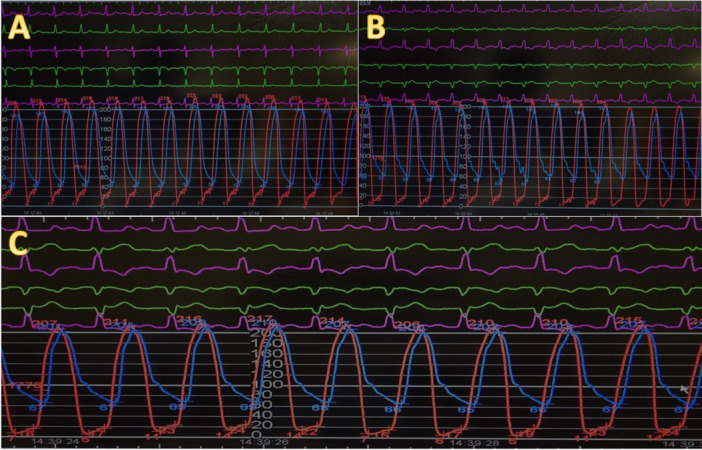
Showing pre and post TAVR invasive Ao/LV pressure measurement for patient 3. (A) Pre TAVR (B) Post TAVR (C) following postdilation using 18 mm nucleus balloon. [Color figure can be viewed at wileyonlinelibrary.com]

**Figure 5:**
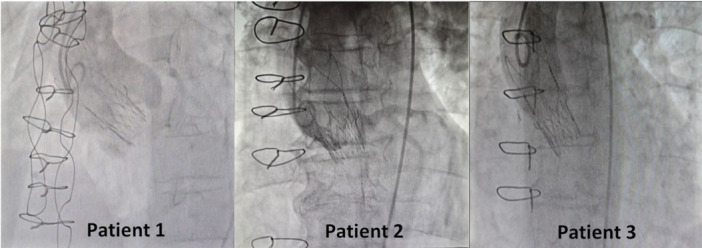
Showing final aortogram post ViV TAVR. [Color figure can be viewed at wileyonlinelibrary.com]

Patients mean age was 75 ± 7 years, 67% female. The mean time from initial surgery to ViV procedure was 18 ± 1 years. All patients were at high surgical risk, mean STS score of 8 ± 7% and mean EuroSCORE II of 17 ± 7% (Table 2). 2/3 of patients had minimalist approach using right common femoral artery (CFA) as the main arterial access (14 F iSleeve sheath) and right radial artery as the secondary arterial access (6 F sheath) whereas Patient 1 required bilateral CFA as vascular accesses (Right CFA as main access and Left CFA as secondary access using 7 F sheath). As part of our standard of practice, after gaining vascular access a pigtail catheter was positioned at the nadir of the non‐coronary cusp (NCC) for root assessment and landmark, the bioprosthetic valve was crossed using an Amplatz Left 1 (AL1) and a straight 0.35 wire which was then exchanged for a Safari wire. Invasive peak‐to‐peak gradients were taken (Figures 2, 3, 4). The mode of pacing for all 3 patients was “pacing on LV wire” at 140 beats per minute (bpm). ViV procedure was successfully completed in all patients and similarly, 2/3 of patients required post‐dilatation due to residual gradient across the TAVR prosthesis and this was achieved while pacing at 180 bpm with 18 mm True balloon for patient 1 and 18 mm Nucleus balloon for patient 3 (Figures 2, 3, 4). Final invasive gradients and aortogram were satisfactory (Figures 2, 3, 4, 5).

**Figure 6:**
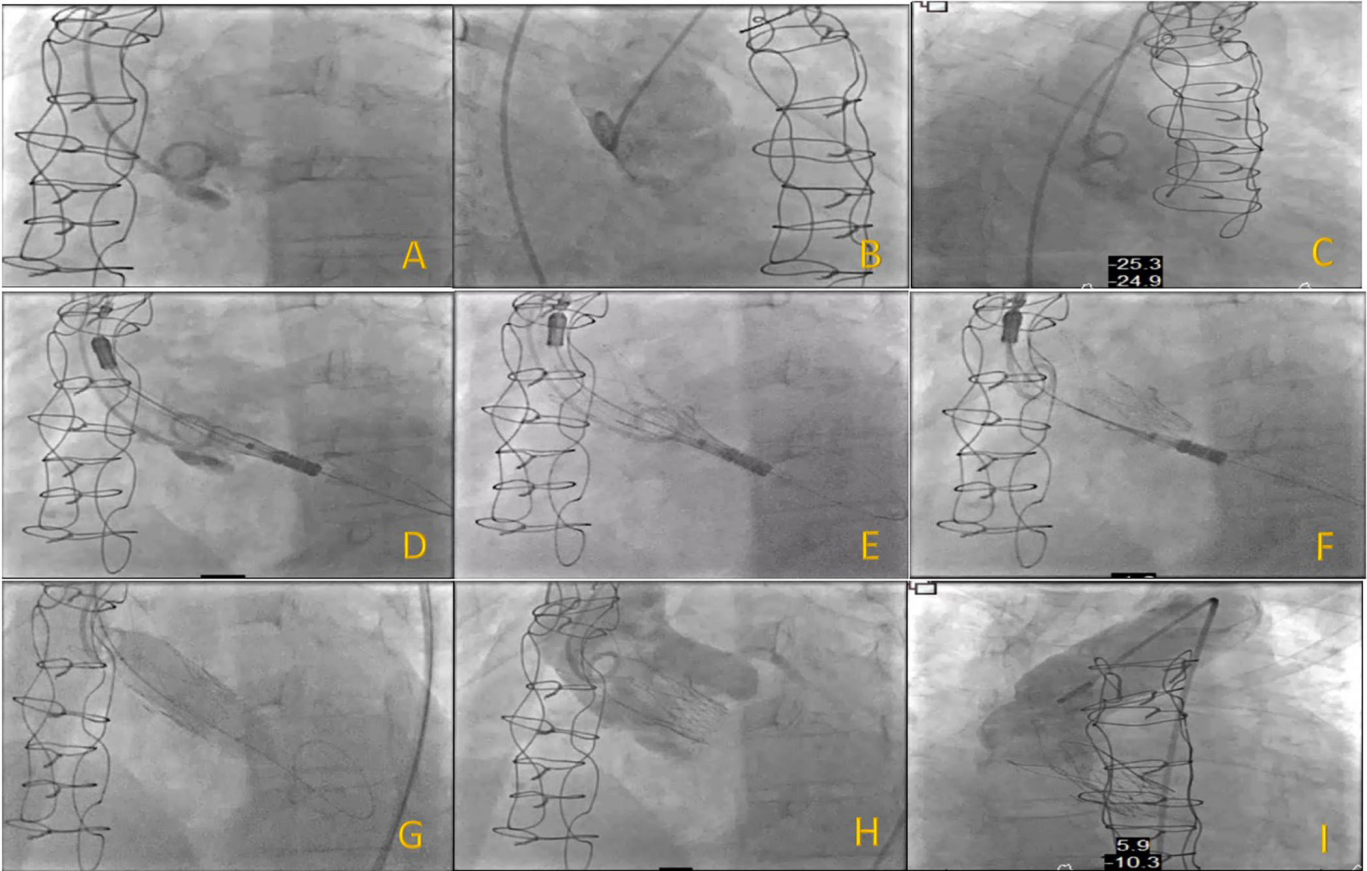
(A–I) Showing implantation steps and technique for patient 1. [Color figure can be viewed at wileyonlinelibrary.com]

Due to anatomic complexity for patients 1 and 2 the procedural technique was modified in the following way: For patient 1—Image acquisitions were obtained in different projections to identify the dissection flap with a view to avoid instrumentation of this area during the procedure, the bioprosthetic valve was crossed in the 3 cusp (co‐planar) view using the J‐tip of 0.35 wire and Amplatz Left 1 (AL 1) catheter, 0.35 wire was exchanged for a Safari wire and the delivery catheter system (DCS) was positioned ensuring commissural alignment in the ascending aorta before crossing the valve. The radio‐opaque marker of the DCS was positioned below the pigtail in the annular plane, and by maintaining the DCS on the outer curve of the aorta step 1 of the deployment was initiated with opening of the stabilization arches. The arch adjacent to the NCC helped to secure the dissection flap and step 2 (full deployment) was performed under rapid pacing‐on‐wire at 140 bpm. In view of residual gradient, postdilation was carried out using 18 mm True Balloon under rapid pacing at 180 bpm. Further image acquisitions (cine) were obtained in different projections to confirm that the chronic Type A aortic dissection had not worsened (Figure 6).

**Figure 7:**
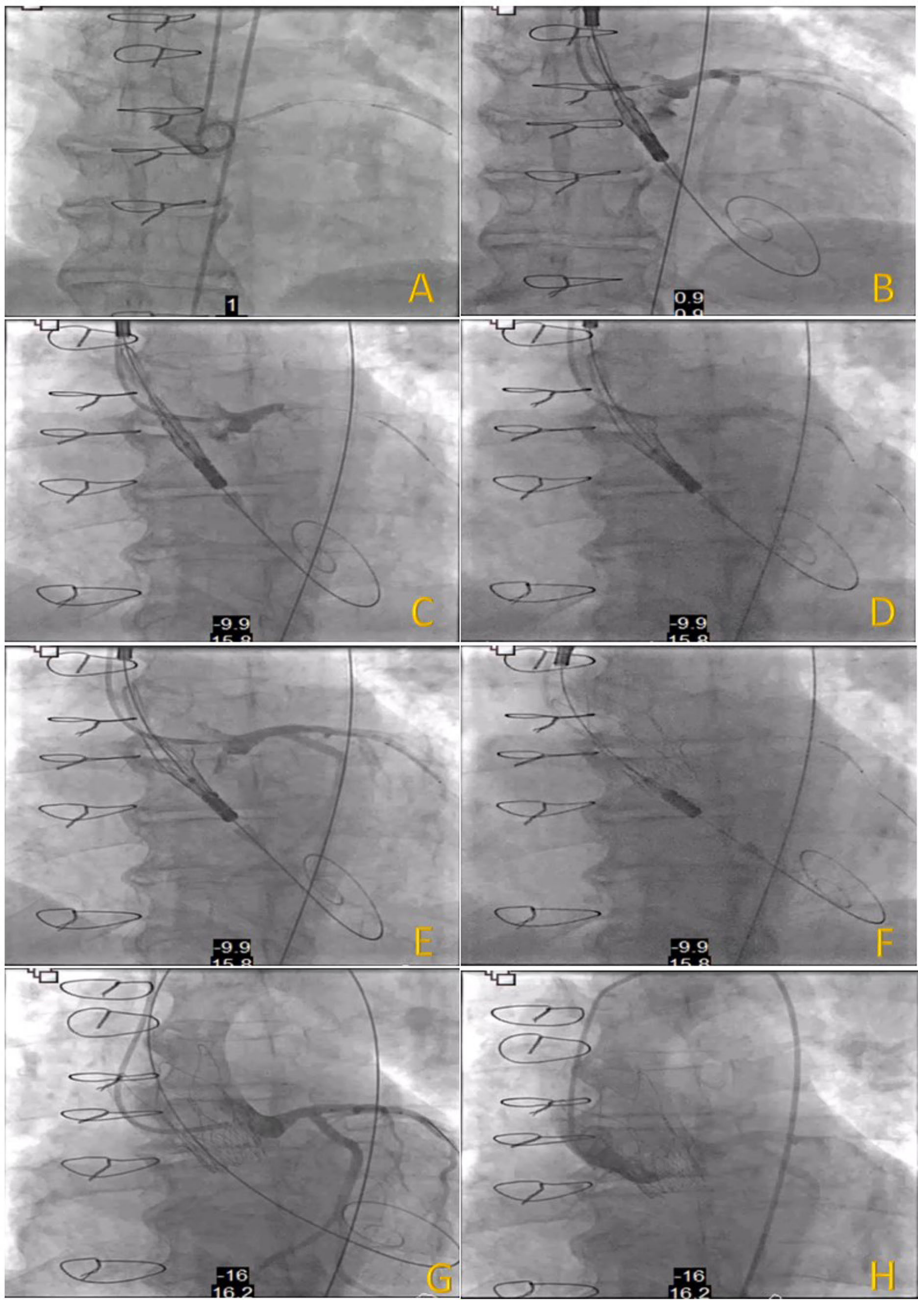
(A–H) Showing implantation steps and technique for Patient 2. [Color figure can be viewed at wileyonlinelibrary.com]

For patient 2—as with every procedure, the annular plane was delineated using a pigtail catheter initially, following this an extra back up 3.5 (EBU 3.5) guide catheter was used to cannulate the left main stem (LMS) artery via the right radial artery, a Sion Blue wire was passed down into the left anterior descending (LAD) artery and a coronary stent (4.0 × 28 mm Xience) was positioned in the proximal part of the LAD with view to perform a Chimney/Snorkel stenting if coronary occlusion occurs after valve deployment. The bioprosthetic valve was crossed in the co‐planar view using the straight 0.35 wire and AL1 catheter, 0.35 wire was exchanged for a Safari wire and the DCS was positioned across the valve as per protocol following commissural alignment. The EBU 3.5 guide was then retracted and used to delineate both the coronary ostia and root using contrast injections. Following this, step 1 of the deployment was initiated with opening of the stabilisation arches, by maintaining a gentle and steady forward tension on the DCS and confirming good position with contrast injection step 2 was carried out under rapid pacing‐on‐wire at 140 bpm. Subsequently, contrast injection revealed that coronary artery patency was maintained therefore, the undeployed coronary stent was cautiously withdrawn and removed along with coronary wire. Final angiography showed no evidence of significant paravalvular regurgitation and presence of TIMI 3 flow within the coronary arteries (Figure 7).

The benefit of choosing this valve platform amongst others is owing to its “top‐down pattern of deployment” unlike other platforms with down‐top pattern thus allowing for gentle and steady forward tension on the DCS during valve deployment especially after step 1 this ensures the upper crowns are hinged on the bioprosthesis chiefly in cases of stentless bioprostheses with absence of visible posts (radiographic markers). More so, this valve platform has a very large cell thus allowing for easy coronary access following its deployment.

Vascular haemostasis were achieved using Perclose Proglides for CFAs and Terumo radial band for right radial arteries. Patient 1 and 2 recovered very quickly and were off i.v diuretics within 24 h following ViV TAVR procedure, all three patients began mobilizing the next day.

There was no major complication, and no more than trivial trans/paravalvular regurgitation on predischarge echocardiogram. There was a clinically significant improvement in renal function following ViV procedure (difference of 12, eGFR 42 ± 3 mL/min/1.73 m² preprocedure to eGFR 54 ± 12 mL/min/1.73 m² postprocedure) however, this was not statistically significant *p* = 0.089. All 3 patients were discharged home within Day 1–3 postprocedure. No patient required permanent pacemaker implantation and there was no stroke or mortality at 30‐day (Table 2). All patients were reviewed at our nurse‐led TAVI clinic after 6 weeks, and reported significant improvement in NYHA functional class along with reduction in symptoms such as breathlessness, fatigue, and ankle swelling.

## 3 Discussion

The use of AN2 for ViV TAVR especially in patients with Shelhigh supra stentless bioprosthetic valve appears to be a feasible and effective option. The minimalist approach, which minimizes the use of sedation, invasive monitoring, and complex procedural steps was associated with low procedural morbidity and favorable early outcomes which agrees with similar approach used in previous study [9]. Patient 1 presented with other challenges including residual chronic type A aortic dissection flap in non‐coronary cusp due to her previous aortic wrap surgery (Figure 1a), large body habitus (BMI 38.7 kg/m²) and Killip class IV. Patient 2 had very low coronary height (Figure 1b) thus required coronary protection using a guide catheter, angioplasty wire and undeployed coronary stent which was positioned prior to valve deployment ready for use in case of any coronary occlusion. Patient 3 had a small annulus (Figure 1c) similar to the other patients however, they were all treated successfully using small size (23 mm) AN2 valve.

The absence of visible posts and stent frame in the failed bioprosthesis did not pose an insurmountable challenge with AN2 platform. These devices which are more flexible and adaptable than balloon‐expandable valves, allow for more precise positioning and easier deployment even within the complex anatomy of stentless valves [10].

Our findings suggest that this approach can be safely performed with excellent short‐term results. However, the long‐term outcomes including durability of ViV procedure, will require further investigation.

## 4 Conclusion

Valve‐in‐valve TAVR using AN2 along with a minimalist approach represents a promising strategy for treating patients with failed Shelhigh supra stentless bioprosthetic aortic valves. This technique offers a great alternative to redo surgery, with favorable procedural outcomes and a low complication rate. Larger, multicentre studies with longer follow‐up are required to better understand the long‐term benefits and limitations of this approach.

## Consent

The author(s) confirm that written consent for submission and publication of this case report including image(s) and associated test has been obtained from the patient in line with the COPE best practice guidelines, and that patients who are being reported on are aware of the possible consequences of this reporting.

## Conflicts of Interest

The authors declare no conflicts of interest.

## Data Availability

Data sharing is not applicable to this article as no new data were created or analyzed in this study.
